# How Does Exercise Improve Implicit Emotion Regulation Ability: Preliminary Evidence of Mind-Body Exercise Intervention Combined With Aerobic Jogging and Mindfulness-Based Yoga

**DOI:** 10.3389/fpsyg.2019.01888

**Published:** 2019-08-27

**Authors:** Yifan Zhang, RuoFan Fu, Li Sun, YuJing Gong, Donghui Tang

**Affiliations:** College of P. E. and Sports, Beijing Normal University, Beijing, China

**Keywords:** mind-body exercise, aerobic jogging, mindfulness-based yoga, implicit emotion regulation ability, aerobic fitness, mindfulness, potential pathway

## Abstract

**Purpose:** The primary aim of the present study is to examine the effect of 8-week mind-body exercise intervention combining aerobic jogging and mindfulness-based yoga on implicit emotion regulation ability. The secondary aim is to explore the specific potential pathways by which the mind-body exercise intervention fosters implicit emotion regulation. This may help us to understand how the key components of exercise intervention contribute to emotional benefits.

**Methods:** Sixty participants were randomly allocated to one of two parallel groups: (1) the intervention group (*n* = 29) and (2) the waitlist control group (*n* = 31). Participants were asked to fill out scales measuring mindfulness and instructed to complete an emotion regulation task to assess implicit emotion regulation ability as well as the PWC 170 Test to evaluate aerobic fitness before and after the intervention.

**Results:** The results of the two-way repeated ANOVA revealed that 8 weeks of intervention improved implicit emotion regulation, mindfulness, and aerobic fitness levels. Path analysis showed that only improved aerobic fitness mediated the intervention effect on implicit emotion regulation ability, controlling for change in negative affect. Notably, the relationship between the effects on implicit emotion regulation ability and aerobic fitness was moderated by improved mindfulness.

**Conclusion:** Eight weeks of mind-body exercise intervention improves implicit emotion regulation ability. The aerobic fitness may be an essential pathway which mediates the efficacy on implicit emotion regulation ability. Furthermore, different components, such as aerobic fitness and mindfulness, may interactively contribute to such emotional benefits.

## Introduction

Emotion regulation is conceptualized as the process by which an individual influences the occurrence, experience, and expression of emotions (Gross, 1998). This process can not only operate in explicit levels but also as an implicit process without the need for conscious supervision or deliberative intentions, and both processes contribute considerably to the effectiveness of emotion regulation as well as mental health (Gross and Thompson, 2007). Literature has emphasized the importance of emotion regulation in determining whether stressful life events result in psychological distress (Sheppes et al., 2015). Specifically, when high stress is perceived, the increase in psychological distress is associated with emotion regulation difficulties (Abravanel and Sinha, 2015). To improve the ability to regulate emotion, several psychological treatments have been developed, such as mindfulness-based cognitive therapy, emotion-focused therapy, and emotion regulation therapy (Mennin et al., 2002; Santor, 2003; Bishop et al., 2004). Additionally, exercise is another complementary intervention that might aid emotion regulation.

Recent research has demonstrated that exercise has salutary effects on emotion regulation ability (Bernstein and McNally, 2016, 2017; Edwards et al., 2017, 2018). However, the specific underlying mechanisms by which varieties of exercise interventions exert their influence on emotion regulation are not clear. One potential explanation for the impact might be aerobic fitness (Lott and Jensen, 2016), which is enhanced by aerobic exercise. Another explanatory pathway might be mindfulness improved by particular exercises, such as yoga or tai chi, which are closely related with emotion regulation. Changes in both aerobic fitness and mindfulness are potential health indicators that might mediate intervention effects on emotion regulation.

### Aerobic Fitness and Emotion Regulation

Recent studies have indicated that habitual aerobic activity and aerobic fitness are associated with emotion regulation and mood benefits (Brown et al., 2013; Giles et al., 2017). Lott and Jensen (2016) implemented a cross-sectional study that indicated that executive control is a mediator of the association between aerobic fitness and emotion regulation. However, the causality cannot be inferred from the temporal precedence of the hypothesized associations in this study. Experimental studies need to examine the effect of aerobic activity on emotional regulation. Previous data from our laboratory provided evidence of causality, indicating that 8 weeks of aerobic exercise fosters the frequency of adaptive emotion regulation strategy and that the improvement of inhibition and switching mediate the effect of 8 weeks of aerobic exercise (Zhang et al., 2017). Regarding the effect on emotion regulation ability, the effect of chronic aerobic exercise has not been investigated but certainly exists because of the strong effect of even concentrated aerobic exercise. For example, evidence from a random control trial showed that individuals doing 30 min of jogging recovered faster than their counterparts who did not exercise after the same negative emotion induction (Edwards et al., 2017). Another random control trial indicated that an acute aerobic exercise facilitates the ability to down-regulate emotion, manifesting attenuating emotion responses to negative emotion cues (Bernstein and McNally, 2016). One potential explanation is that the emotion regulation benefits associated with acute aerobic exercise might be due to the improvement of executive function, in view of its essential role in emotion regulation (Ochsner and Gross, 2007). Given chronic aerobic exercise improves both aerobic fitness and emotion regulation ability, we assume that the improvement of aerobic fitness might mediate the effect on emotion regulation ability.

### Mindfulness and Emotion Regulation

Mindfulness is conceptualized as a state of nonjudgmental attentiveness to and awareness of moment-to-moment experiences (Bishop et al., 2004) and is associated with decreased negatively biased cognition and explicit and implicit emotion processing (Kiken and Shook, 2012; Greenberg and Meiran, 2014). A neuroscience study suggested that the level of mindfulness is associated with increased prefrontal cortical activation and reduced bilateral amygdala activity during an affect-cue task (Creswell et al., 2007). This finding indicated that individuals with a high level of mindfulness are more likely to activate the cognitive control system to regulate emotional responses as the top-down approach (Ochsner and Gross, 2005; Creswell et al., 2007). In addition, mindfulness is enhanced by mindfulness-based training, with the consequence of improved emotion regulation (Guendelman et al., 2017). In the clinic, mindfulness has been integrated into the treatment programs of emotional disorders such as anxiety and depression disorder (Kabatzinn, 1982; Riemann et al., 2016). In sport psychology, a mindfulness-based program has been specifically designed for athletic performance enhancement (Gardner and Moore, 2005) *via* reductions in negative coping as well as an improved capacity to regulate negative emotions in competition (Josefsson et al., 2017). In exercise psychology, a growing body of empirical evidence has showed the unique efficacy of the type of mindfulness-based exercise on emotion regulation (Menezes et al., 2015).

To explain the effect, Garland et al. (2009) presented a causal model explicating the mechanism of mindfulness meditation action. According to the mindful coping model, mindfulness-based training made it easier for individuals to decenter to the mode of mindfulness, increasing attentional flexibility and broadening awareness, which played an important role in positively reappraising by attributing positive meaning to the event (Garland et al., 2009). Furthermore, researchers provided more evidence to verify the correspondence that increases in mindfulness are significantly correlated with reductions in avoidance and rumination during mindfulness-based intervention (Kumar et al., 2008). Against this backdrop, we assume the improved mindfulness might also explain emotion regulation ability improved in intervention.

### Present Study

In the present study, we assume exercise intervention is just a medium or tool to contain different kinds of key components like aerobic fitness, mindfulness, and motor skills. The keys to underlie efficacy are “the active ingredients” in exercise intervention, not the intervention itself. Therefore, we are interested in “the active ingredients” in exercise intervention and evaluation of potential pathways that may underlie the effect on implicit emotion regulation. We attempt to deconstruct the role of different components of exercise to better understand their respective or/and interactive contributions to the effect.

To address this, we learned from previous study to design the mind-body exercise intervention (Alderman et al., 2016). The present intervention consisted of aerobic intervention and mindfulness intervention. In aerobic intervention, jogging was used, because of its simple component of aerobic. In mindfulness intervention, a specially designed mindfulness-based yoga training was applied, which maximized emphasis on the component of meditation in yoga practice, aiming to cultivate the state of mindfulness and minimized emphasis on intensity and motor skills, in order to maintain heart rate (not elevated) during yoga practice. All these designs tried to reduce confounding effects.

Although implicit emotion regulation playing an important role in emotion regulation is believed to be more representative of daily life requirements (Gross and Thompson, 2007; Rothermund, 2011), the effect of exercise on implicit emotion regulation has not been investigated. Based on these considerations, the primary aim of the present study is to examine the effect of mind-body exercise intervention on implicit emotion regulation ability. The secondary aim is to explore the specific potential pathways. Put concretely, do increases in aerobic fitness and/or mindfulness scores mediate the intervention effect? Do increases in aerobic fitness and mindfulness scores interactively contribute to the intervention effect?

### Methods

#### Participants

Sixty female postgraduates were recruited from a university in Haidian District, Beijing. *A priori* G-Power analysis was used by G-Power 3.1 program, with a significance level *α* = 0.05, required power (1 − *β*) = 0.8. In general, benefits of chronic exercise are no worse than acute exercise. Therefore, the *η*^2^ expected from the study which tests the reduction of negative affect after an acute aerobic exercise during affective induction was 0.22 (Smith, 2013). The sample of 29 in exercise group and 31 in control group resulted in sufficient participation for this study.

All participants recruited were screened by completing several surveys to ensure that they met the inclusion criteria: (1) no previous training in or current practice of meditation; (2) low level of exercise experience [participants who responded with 1 or 2 (on a 1–5 scale) to all of the following statements were included: “What intensity of exercise do you usually participate in?,” “How long do you exercise when you participate in the above-mentioned exercise,” and “How many times have you participated in the above-mentioned exercise this month”]; (3) no anxiety disorders, defined as a score < 50 points on the Self-Rating Anxiety Scale-Chinese version (Duan and Sheng, 2012); and (4) no use of any medications that may alter mood. Of note is that additional surveys measured during recruitment to ensure that baseline psychological parameters and experience of meditation and exercise were homogeneous in the two groups. All participants provided written consent, and the protocol was approved by the institutional review board of *College of P.E and sports, Beijing Normal University*.

### Measure

#### Emotion Regulation Task

The emotion regulation task was modified by previously reported emotion regulation paradigms (Gross and John, 2003) that assessed implicit emotion regulation ability by computing each participant’s average magnitude of reduction in negative affect while responding to negative images with the manipulation was meant to induce implicit emotional down-regulation, compared to responding to matched negative images without manipulation. Forty pictures selected from the International Affective Pictures System (IAPS) (Lang et al., 1999) were divided into four matched blocks (2 blocks × 2 times, no significant difference in the valence of each block, according to the results of the pre-experiment in another sample of 32 female postgraduates, *F* = 0.169, *p* = 0.917 > 0.05). All stimuli were presented *via* E-prime (ver. 1.1).

The emotion regulation task consisted of two blocks: (1) watching without engaging in emotion regulation and (2) watching with subliminal goal priming. In each block, 10 pictures were presented. During subliminal goal priming, no subliminal priming was mentioned, and participants were asked to evaluate the presented adjectives (Chi and Lin, 2003) by pressing the “positive” or “negative” key on the keyboard. After the false task, the masked affective priming was closely modeled (Custers and Aarts, 2007). The priming words were proven to induce spontaneous emotional down-regulation (Du et al., 2014). At the end of each picture, a nine-point scale was used to measure subjective feeling from 1 (extremely unpleasant) to 9 (neutral).

#### Assessment of Aerobic Fitness

The Physical Working Capability 170 (PWC 170) test was used to assess improved aerobic fitness as a result of aerobic exercise intervention. For this test, participants cycled on a Monark834 cycle ergometer for two stages. For the first stage, each participant began at 30 W of resistance and maintained the work rate until her heart rate remained stable (recorded continuously every 5 s by the Polar RS800 heart watch). According to previous studies, the resistance in the second stage ranged from 66 to 88 W, based on the stable heart rate in the first stage to ensure the validity of the test. The literature has shown that the PWC170 test was just as valid a predictor of VO_2_ max (*r* = 0.84) (Boreham et al., 1990).

#### Assessment of Mindfulness

The Mindful Attention Awareness Scale (MAAS; Brown and Ryan, 2003) is a six-item self-report measure assessing improved mindfulness as a result of yoga intervention. The MASS has been shown to have excellent internal consistency across a range of samples (Cronbach’s *α* = 0.84). The Chinese version of the MASS has also shown great psychometric properties (Deng et al., 2011). Previous studies have shown a larger improvement of MASS scores with more intense practice and supported using the change of MASS scores as a “surrogate measure” that can reflect the intensity of mindfulness practice (Brown and Ryan, 2003).

#### Additional Assessment

Several surveys were employed to evaluate baseline demographic, behavioral, and psychological characteristics across the two groups. Briefly, the additional baseline parameters that were assessed included energy expenditure estimates (METs) per week (*via* the International Physical Activity Questionnaire-Chinese version) (Qu and Li, 2004), body fat percentage (*via* InBody human body composition analyzer), and anxiety (*via* the Self-Rating Anxiety Scale-Chinese version) (Duan and Sheng, 2012). Due to the potential effects of mood-congruent emotion processing bias (Beck and Aaron, 2008), negative affect was also assessed as a potential covariate (*via* the Positive and Negative Affect Scale-Chinese version) (Huang et al., 2003) before and after the intervention.

#### Mind-body Exercise Intervention

The whole intervention sessions were provided three times a week over an 8-week timeframe. Each of aerobic sessions lasted for 40 min and yoga sessions for 60 min. Two different interventions were provided alternately.

In the aerobic exercise session, participants jogged in three groups on a playground for 30 min and then walked and stretched to actively relax for 10 min. The intensity of jogging was set at moderate (60–70% of the predicted maximal heart rate) and monitored by portable heart watches. Each time, one participant was randomly selected from each group, and each of these three participants was asked to lead her own group to better control the heart rates of all participants by adjusting their running speeds based on her own heart rate.

Yoga training sessions followed a consistent sequence that included: (1) 10 min of breathing-regulation practice and 5 min of focused-attention meditation in a comfortable, cross-legged, seated position, (2) 30 min of postures and movement sequences practice integrated by mindfulness prompts, and (3) 10 min of mindfulness meditation and 5 min of loving-kindness meditation. This session was strictly designed using the following aspects.

##### Conscious Breathing

Different conscious practices of altering breathing patterns were conducted during breathing regulation, movement sequences, and meditation. In breathing regulation, the focus was simply on cultivating breath awareness and conscious breath regulation, including the depth and frequency of breathing. In movement sequences, breathing should precisely coordinate with movement. In focused attention meditation, breathing served as an object of attention.

##### A Combination of Postures and Movement Sequences

A series of pulling movements designed to warm up the extremities and spine were conducted before practicing sitting and standing postures. This was followed by seated postures practice, including forward bends, back arches and twists. Then, a series of spine releases were done to relax. As participants became more competent with practice, a series of standing postures were conducted to fully activate the nervous system and muscles of the whole body. Finally, participants lay on the floor and relaxed to calm down and restore their bodies.

##### Meditation

Focused-attention meditation was designed as the starting point to improve attention. Due to the consequences of focused attention, mindfulness meditation was easily conducted to cultivate mindfulness (observing without judgment). The session concluded with loving-kindness meditation, which involved showing compassion, appreciation, and acceptance of the self and others.

### Procedure

Participants were randomly allocated to one of two parallel groups: (1) the intervention group and (2) the waitlist observational control group. All outcomes were collected at the baseline, 9 weeks (post intervention); in addition, pre- and post-assessments were identical and arranged at the same time of day for each individual to control the potential impact of biological rhythm. Assessments were conducted as follows in a consistent sequence: on arrival at the laboratory, participants were asked to fill out scales measuring mindfulness and negative affect levels. Then, participants were instructed to complete the emotion regulation task to assess implicit emotion regulation ability. After this, the Physical Working Capability 170 Test was conducted to evaluate aerobic fitness, and acute exercise potentially also helped to ease the negative impact of the previous affect-induction task.

#### Data Analytic Strategy

A 2 (Group: EI, WL) × 2 (Time: Pre, Post) repeated-measures analysis of variance (ANOVA) was run in SPSS version 20 to examine the effects on implicit emotion regulation ability, mindfulness, aerobic fitness, and negative affect.

Based on the hypothesis, improvement of health indicators not only physically but also mentally may mediate intervention effects on implicit emotion regulation ability. Path analyses were run in Mplus version 7.4 (Muthén and Muthén, 1998–2015) to examine the potential explanatory pathways with (1) the independent variable (expressed as two “dummy variables”: exercise as Group 1, control as Group 2); (2) the mediating variable (improved aerobic fitness and mindfulness); (3) the dependent variable (improved implicit emotion regulation ability); and (4) the covariate (change in negative affect). Through bias-corrected nonparametric bootstrapping statistical analyses, data were re-sampled 1,000 times, and confidence intervals (BCIs) of the indirect and direct effects were set at 95%, with an *α* level of 0.05 (Preacher and Hayes, 2008).

The hierarchical multiple regression analysis was used to estimate individual and/or interaction contributions to improved implicit emotion regulation ability accrued by intervention-related factors, which entailed the following steps: (1) interaction variables were computed by multiplying the predictors (improved mindfulness × improved aerobic fitness). (2) Hierarchical multiple regression analyses with the forced entry method were run for the prediction of improved implicit emotion regulation ability. Potential covariate (change in negative affect) was entered in the first block for statistical control, while the factors related to intervention (improved mindfulness and aerobic fitness) were entered in the second block and the interaction term in the third block. (3) If the interaction term in the third block added to the model, a significant percentage of variance explained, *post hoc* analyses were run by simple slope testing (Aiken and West, 1991).

## Results

Descriptive characteristics and homogeneity test for baseline are displayed in Table 1, showing that there were no statistically significant differences in baseline parameters.

**Table 1:** Baseline characteristics and homogeneity test of the analyzed sample.

Variable	Group		
	Exercise intervention (*n* = 29)	Waitlist control (*n* = 31)	*p*
Age	23.07 (1.51)	23.34 (1.52)	0.33
Body fat percentage	25.25 (4.39)	25.02 (4.36)	0.85
Implicit emotion regulation ability	−1.20 (1.45)	−1.09 (1.26)	0.76
Aerobic fitness	1.69 (0.33)	1.58 (0.38)	0.26
Mindfulness	3.48 (0.64)	3.29 (0.68)	0.28
Negative affect	2.79 (0.59)	2.73 (0.48)	0.68
Anxiety	40.17 (5.78)	40.84 (6.61)	0.68
Energy expenditure estimates	888.79 (620.08)	953.13 (620.66)	0.69

*All values presented as mean (standard deviation). p’s calculated via independent samples t-test*.

A 2 (Group: EI, WL) × 2 (Time: Pre, Post) repeated-measures analysis of variance was conducted on implicit emotion regulation ability, which indicated a main effect of time (*F*_1,59_ = 4.75, *p* < 0.05, $\eta_{p}^{2}$ = 0.076), no effect of group (*F*_1,59_ = 1.558, *p* > 0.05, $\eta_{p}^{2}$ = 0.026), and a significant interaction between group and time (*F*_1,59_ = 7.289, *p* < 0.01, $\eta_{p}^{2}$ = 0.112). Follow-up within-group *t*-tests yielded pre-to-post-exercise increases in implicit emotion regulation ability (*t* = −2.474, *p* < 0.05) and no change for control group (*t* = 0.889, *p* > 0.05).

For aerobic fitness, the results showed a main effect of time (*F*_1,59_ = 55.276, *p* < 0.01, $\eta_{p}^{2}$ = 0.488), and an effect of group (*F*_1,59_ = 6.969, *p* < 0.05, $\eta_{p}^{2}$ = 0.107); furthermore, the interaction between time and group was significant (*F*_1,59_ = 57.123, *p* < 0.01, $\eta_{p}^{2}$ = 0.496). Follow-up within-group *t*-tests yielded pre-to-post-exercise increases in aerobic fitness (*t* = −7.304, *p* < 0.000) and no change for control group (*t* = 0.506, *p* > 0.05).

A 2 (Group: EI, WL) × 2 (Time: Pre, Post) repeated ANOVA showed a main effect of time (*F*_1,59_ = 19.891, *p* < 0.01, $\eta_{p}^{2}$ = 0.255) and group (*F*_1,59_ = 5.299, *p* < 0.05, $\eta_{p}^{2}$ = 0.084), and a significant interaction between group and time for mindfulness (*F*_1,59_ = 20.301, *p* < 0.01, $\eta_{p}^{2}$ = 0.259). Follow-up within-group *t*-tests yielded pre-to-post-exercise increases in mindfulness (*t* = −4.446, *p* < 0.000) and no change for control group (*t* = 0.105, *p* > 0.05).

As for negative affect, there was a main effect of time (*F*_1,59_ = 13.822, *p* < 0.01, $\eta_{p}^{2}$ = 0.192), no effect of group (*F*_1,59_ = 0.512, *p* > 0.05, $\eta_{p}^{2}$ = 0.009), and a significant interaction between group and time (*F*_1,59_ = 4.231, *p* < 0.05, $\eta_{p}^{2}$ = 0.068). Follow-up within-group *t*-tests yielded pre-to-post-exercise decreases in negative affect (*t* = 3.498, *p* < 0.000) and no change for control group (*t* = 1.425, *p* > 0.05).

In the hypothesized model, two indirect pathways were included. The results showed that only improved aerobic fitness, but not mindfulness, was found to significantly mediate the effect of participating in intervention versus control on improved implicit emotion ability, as indicated by bootstrapped 95% confidence intervals that did not contain zero (*αβ* = −0.411, 95% BCI = −0.79 to −0.143). In addition, the path coefficient for the direct effect of group on improved implicit emotion regulation controlling for two mediators and covariate was not significant. Standardized coefficients, bootstrapped 95% confidence intervals (BCIs), and standard errors of direct effects are reported in Table 2.

**Table 2:** Summary of direct effects of mediation model.

	Estimate	S.E.	Lower BCL	Upper BCL
Group→improved implicit emotion ability	0.109	0.239	−0.304	0.655
Group→improved aerobic fitness	−**0.704**	**0.100**	−**0.922**	−**0.524**
Group→improved mindfulness	−**0.509**	**0.120**	−**0.754**	−**0.283**
Improved aerobic fitness→improved implicit emotion ability	**0.584**	**0.218**	**0.166**	**1.056**
Improved mindfulness→improved implicit emotion ability	0.041	0.175	−0.304	0.390

*S.E., standard error; BCI, bootstrapped confidence interval*.

**Figure 1:**
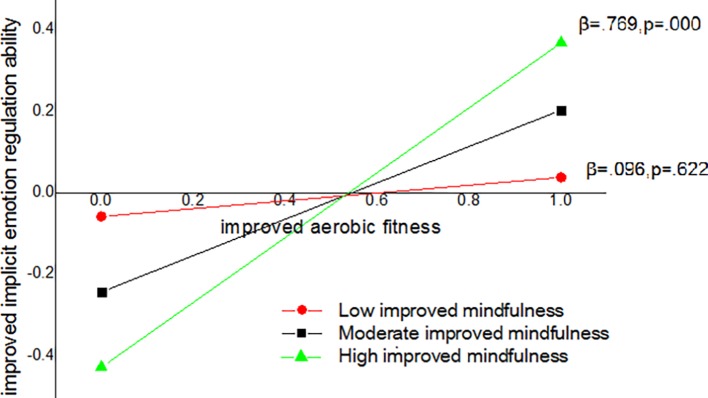
Improved implicit emotion regulation ability as a function of improved aerobic fitness in lower and higher improved mindfulness: a moderated prediction.

Table 3 shows significant results of hierarchical regression analyses. With regard to the variance explained, improved implicit emotion ability was positively predicted by improved aerobic fitness, contributed to the prediction with 26.8%. The introduction of the interaction term to the model added a small but significant percentage of variance explained (8.9%), indicating the presence of a moderated effect. A *post hoc* analysis was conducted with change in negative affect as a covariate, improved aerobic fitness as a predictor of improved implicit emotion regulation ability, and improved mindfulness (high level, moderate level, and low level) as a moderator. Only at a high level and a moderate level of improved mindfulness could improvements in implicit emotion regulation ability be predicted by improved aerobic fitness (Figure 1).

**Table 3:** Hierarchical multiple regression analysis to predict improved implicit emotion ability.

Predictors	Beta (std)	*T*	*p*
Block 1			
Change in negative affect	−0.134	−1.028	0.308
▵*R*^2^		0.018	
Block 2			
Improved aerobic fitness	**0.519**	**4.104**	**0.000**
Improved mindfulness	**0.018**	**0.127**	**0.899**
▵*R*^2^		0.268	
Block 3			
Improved aerobic fitness × Improved mindfulness	**0.359**	**2.793**	**0.007**
▵*R*^2^		0.089	
Total *R*^2^ = 0.375			
Adjusted total *R*^2^ = 0.329			

*VIF statistics ranged between 1.0 and 1.7, thus allowing to exclude problems of multicollinearity*.

## Discussion

The primary aim of the present study was to examine the effect of mind-body exercise intervention on implicit emotion regulation. To address this question, we investigated the changes in implicit ability to down-regulate negative affect before and after 8 weeks of intervention or control. The results of two-way repeated ANOVA revealed that only participants in the intervention group displayed significant improvement in implicit emotion regulation ability. Apart from one study that found the brief mindfulness manipulation fosters implicit emotion regulatory processes (Remmers et al., 2016), few studies have examined the effect on implicit emotion regulation, especially with long-term intervention. Thus, the present study extends previous empirical research on the benefits of exercise intervention on implicit emotion regulation and demonstrates that 8 weeks of mind-body exercise intervention improves the implicit emotion regulation ability.

The secondary aim was to explore potential mechanisms of the effect on implicit emotion regulation ability. Based on the premise, confirmed by our results, that an 8-week intervention improved aerobic fitness, mindfulness, and negative affect, path analyses indicated that only improved aerobic fitness mediated the intervention effect on implicit emotion regulation ability. In line with the neuronal plasticity hypothesis, the improvement of aerobic fitness may result in observable concomitant structural and functional changes in regions of prefrontal and parietal cortices (Colcombe et al., 2004; Hillman et al., 2008). These brain regions are regarded as important regions for realizing emotion regulation; for example, the ventral-anterior cingulate cortex (vACC) and ventro-medial prefrontal cortex (vMPFC) are involved in implicit emotion regulation (Kohn et al., 2014; Etkin et al., 2015), and for realizing cognitive control, which is, in turn, hypothesized to be the essential mechanism of top-down emotion regulation (Ochsner and Gross, 2007). Therefore, exercise intervention may directly model brain regions involved in emotion regulation through improved aerobic fitness but may also be involved in cognitive control, indirectly affecting emotion regulation. Unexpectedly, improved mindfulness did not mediate the intervention effect. The present findings fit with the mindful emotion regulation model, not the mindful coping model (Chambers et al., 2009). Mindfulness is not a process of cognitive reappraisal but a unique emotion regulation strategy exerted by the state of mindfulness. In contrast to the cognitive reappraisal strategy, mindful emotion regulation is mainly involved in the bottom-up approach (Chambers et al., 2009; Farb et al., 2012; Chiesa et al., 2013). No indirect effect of improved mindfulness was found in the present results, since the presented priming stimulus might mostly relate to the cognitive reappraisal strategy involving the top-down mechanism. In addition, as the medial prefrontal cortex is also involved in dopaminergic circuits, aerobic exercise may continuously stimulate the dopaminergic circuits through impacting related brain regions, with a consequence of better resistance to negative emotional influence (Codella et al., 2016).

Notably, the relationship between the effect of exercise intervention on implicit emotion regulation ability and aerobic fitness was moderated by improved mindfulness, and improved aerobic fitness could predict greater improvement of implicit emotion regulation ability, especially with higher levels of improved mindfulness in individuals. Although improved mindfulness could not predict improvement of implicit emotion regulation ability directly, exercise-involved mindfulness practice might amplify the effectiveness of simple aerobic exercise to improve emotion regulation, which indicated considerable advantages of a mixed exercise intervention. To our knowledge, studies have stated that exercise interventions involving more effective components, such as aerobic and cognitively engaging exercise, are more beneficial to cognition than simple aerobic exercise without thinking (Colcombe and Kramer, 2003; Best, 2010; Diamond, 2015). This view is also extended from cognitive to emotion regulation by the present empirical results.

The present experiment inevitably has limitations. First, we mainly focused on aerobic fitness as the only relevant outcome of aerobic jogging that may be associated with emotion regulation and on mindfulness as the only relevant outcome of mindfulness-based yoga that may be associated with emotion regulation. Although other outcomes of aerobic exercise and yoga may be not the key active ingredients that underlie the efficacy and some of them are difficult to assess, these outcomes may be potentially linked to emotion regulation through different pathways, such as motor fitness improved by postures, movement-sequence training, and self-compassion, acceptance cultivated by meditation skills (Laura et al., 2015), and these outcomes might be neglected.

In addition, as we have discussed before, mindful emotion regulation may be an implicit strategy involved in the bottom-up approach; however, the paradigm task in the present study was used to mainly evaluate the ability to top-down regulate emotion in the condition of subliminal goal priming. Although the top-down and bottom-up approaches are both comparably effective in emotion regulation, they may regulate negative emotion through different psychological mechanisms, with the consequence that we did not discover the pathway *via* mindfulness and thereby might have neglected its contribution to implicit emotion regulation ability. Future research in this domain would do well to address these limitations.

Finally, as a previous study has shown that mental and physical training, similar to present intervention, decreases maladaptive emotion regulation strategy (ruminative) and negative affect (Alderman et al., 2016). Even though the priming stimuli mainly involved one specific adaptive strategy and we also controlled the change in negative affect, the confounding variables may still exist.

These caveats notwithstanding, the present study sheds important new light on the interface between exercise and implicit emotion regulation. The results showed that exercise improves emotion regulation ability not only at the explicit level but also at the implicit level. However, the sample of present study and effective size for main dependent variable are relatively small, which means the magnitude of the difference is low. The reason might be the duration of intervention is relatively short, while longer intervention treatment such as 12-week interventions may show greater effects. Therefore, we should cautiously interpret the present results and seek a larger sample in future studies.

A further novelty of the study is that it furthers understanding of the potential pathways by which exercise intervention including different components fosters implicit emotion regulation. This may be useful in targeting interventions for those who want to benefit most. Although the findings from the present pilot study are preliminary, one potential mechanism by which exercise exhibits its beneficial effect might be aerobic fitness. For people who desire the benefits of emotional regulation from exercise, the components of exercise including aerobic are absolutely necessary, and the components could be enriched with more cognitive engagement like mindfulness at people’s earliest convenience.

## Data Availability

The datasets generated for this study are available on request to the corresponding author.

## Ethics Statement

The studies involving human participants were reviewed and approved by The Institutional Review Board of School of PE And Sports, Beijing Normal University. The patients/participants provided their written informed consent to participate in this study.

## Author Contributions

YZ conceived and designed the work, drafted and led the work to the submission. In addition, he mainly acquired data and analyzed it. RF, LS and YG contributed to the revision. DT helped to perform the analysis with constructive discussions and approved the final version.

### Conflict of Interest Statement

The authors declare that the research was conducted in the absence of any commercial or financial relationships that could be construed as a potential conflict of interest.
